# Possible Role of Corollary Discharge in Lack of Dyspnea in Patients With COVID-19 Disease

**DOI:** 10.3389/fphys.2021.719166

**Published:** 2021-08-16

**Authors:** Eduardo Luis De Vito

**Affiliations:** Department of Pneumology and Pulmonary Laboratory, Institute of Medical Research, University of Buenos Aires, Buenos Aires, Argentina

**Keywords:** COVID-19, dyspnea, corollary discharge, neuromuscular dissociation, respiratory muscles, respiratory distress syndrome, respiratory mechanics

A relevant feature of COVID-19 disease is that the lack of dyspnea is recognized in the most critical cases. The subjects who were intubated and ventilated exhibited tachypnea and tachycardia (Al-Omari et al., 2020; Guan et al., 2020; Li et al., 2020). In a retrospective study, dyspnea and tightness in the chest were much more common in deceased patients (Chen et al., 2020). Moreover, it was one of the associated predictors of severe disease and death (Kaeuffer et al., 2020).

The absence of dyspnea can be attributed to a direct neurotoxic impact of the virus and a general response caused within the infectious context (Bertran Recasens et al., 2020). Nouri-Vaskeh et al. (2020) argue that two mechanisms can explain the decreased cognition of dyspnea in COVID-19 disease. They are either the direct invasion of SARS-CoV-2 into Angiotensin Converting Enzyme-2 (ACE-2) expressing brain cells in the limbic system (especially the insular area) or through the indirect toxic effect of the cytokine storm on the corticolimbic network, which plays the main role in expressing the perception of dyspnea (Abernethy and Wheeler, 2008; Parshall et al., 2012). The carotid body could be a site of SARS-CoV-2 invasion due to the local expression of its ACE2 receptor (Porzionato et al., 2020). The influence of the nasal flow on the modulation of breathing has recently been described (Tantirigama et al., 2020). This result may be relevant as anosmia has been reported in association with COVID-19 disease.

Dyspnea is the main experience related to the avoidance of survival threats; it is a multidimensional construct of breathing sensations with qualitative, quantitative, and descriptive components (Abernethy and Wheeler, 2008; Parshall et al., 2012; Kaeuffer et al., 2020). The pathophysiological mechanisms, rather than pain, are complex and can coexist (Nishino, 2011). Evaluating dyspnea in the context of COVID-19 disease is difficult, a scenario far removed from the elegant laboratory studies. A proper assessment depends on self-reporting, and so it is imperative to ask open-ended questions. Leading questions aimed at seeking support can be tricky (Tobin, 2020).

The breathing mechanics of patients with COVID-19 seem to have certain peculiarities. Unfortunately, however, information is scarce, incomplete, and disorganized (Mezidi et al., 2020). Gattinoni et al. (2020) proposed two time-dependent chronological phenotypes, namely, type L, which is characterized by low elasticity, low ventilation-to-perfusion ratio, low lung weight, and low recruitment capacity; and type H, characterized by high elasticity, high right-to-left shunt, high lung weight, and high capacity recruits. In addition, various findings in connection with changes in the pulmonary circulation (loss of lung perfusion regulation) are characterized (Picariello et al., 2020; Potus et al., 2020), making it perhaps in vain to recognize type L and type H (Mahjoub et al., 2020). Other authors suspect that adult respiratory distress syndrome (ARDS), in connection with SARS-CoV-2, may not be as atypical as has been suspected (Baedorf Kassis et al., 2021). It appears that the stages are not as dichotomous as suggested above.

To understand the absence of dyspnea in COVID-19, the main focus is on phenotypes showing severe hypoxia and near-normal respiratory system compliance. Despite the mild to severe ARDS during COVID-19 pneumonia, the respiratory system (transpulmonary compliance and driving pressure) was reported pseudonormal (Bhatraju et al., 2020; Viola et al., 2020).

The concepts of corollary discharge (CD) and efferent–afferent dissociation (also called length–tension inappropriateness, neuro ventilatory dissociation, afferent mismatch, neuromechanical uncoupling, or neuromuscular dissociation; Parshall et al., 2012) could explain the absence of dyspnea in COVID-19 disease. Neurophysiological studies have discovered the CD circuits and proposed a functional taxonomic classification through the animal kingdom. The CD is a ubiquitous strategy to route copies of movement commands to sensory structures and influence sensory processing in myriad ways (Crapse and Sommer, 2008). The concept of a CD is the most widely accepted hypothesis used to explain the origin of the inspiratory effort sensation (el-Manshawi et al., 1986) and dyspnea (Spengler et al., 1998; Booth and Dudgeon, 2006; Okada, 2021).

Animal studies demonstrated that a copy of respiratory output is transmitted to the mesencephalon and thalamus (Chen et al., 1990, 1992). Similar to skeletal muscles, the difference in signal between the corollary and the afferent discharge is automatically used to produce a sensation (Matthews, 1982).

When the motor command sends an outgoing command to the respiratory muscles, a copy is sent to the sensory cortex (efferent copies or efference copy signal). The sensory cortex also receives information about events from the chest and respiratory muscles and processes the information (Okada, 2021). When the copy signal is received, the sensory cortex adapts accordingly in order to minimize, eliminate, or compensate for the sensory consequences of the movement (Crapse and Sommer, 2008). Due to this general strategy, breathing under normal conditions (and within certain ventilation limits) is an unconscious process (Crapse and Sommer, 2008). If the demands are fully met, there is no awareness of outgoing motor command, nor is dyspnea experienced. On the other hand, if the two “letters” are different (such as muscle weakness, increased mechanical load, and lungs stiffness), it will lead to a conscious perception of the motor command.

The CD for respiratory sensation is reminiscent of the original concept of Campbell and Howell (1963). They suggested that an imbalance in the relationship between tension and displacement in respiratory muscle is the neurophysiological mechanism causing dyspnea. This neuromechanical dissociation among outgoing (efferent) motor signals to the respiratory muscles and incoming (afferent) information is a key element of the idea of dyspnea. The CD is an updated and revised version of this pioneering work.

The greater the separation among the outgoing and incoming signals, the higher the intensity of the shortness of breath. In COVID-19 disease with respiratory failure, the relationship between the absolute value of loading and its duration and the relative magnitude of the load compared with the maximum muscle strength seems to be reasonable.

It is plausible to assume that the prevalence and intensity of dyspnea depend on the interaction between the severity of the infection, the role of the host response, physiological reserve and accompanying diseases, and the time between the onset and hospitalization (Gattinoni et al., 2020). Hypoxemia is one of the other reasons for shortness of breath and is commonly responsive to oxygen therapy, mechanical ventilation, and pronation, especially in L patients (Gattinoni et al., 2020). However, dyspnea is not necessarily related to hypoxia but is more closely associated with inspiratory drive and mechanical alterations.

Why is there a mismatch in many other lung diseases but no mismatch in COVID-19?

Since the COVID-19 lung disease shows a relative preservation of respiratory mechanics compared with other acute lung diseases (such as the classic ARDS), the hypoxemia is due to the occupancy of the alveoli, which leads to low ventilation/perfusion ($\overset{∙}{\text{V}}$/$\overset{∙}{\text{Q}}$) ratio units; however, pulmonary vascular dysregulation (lack of physiological vasoconstriction in these areas with low $\overset{∙}{\text{V}}$/$\overset{∙}{\text{Q}}$), which causes only insignificant changes in the respiratory mechanics, seems to play an essential role. Once again, neuromechanical dissociation occurs when there is a mismatch between the outgoing (efferent) motor signals to the respiratory muscles and the incoming (afferent) information. If information from chemoreceptors (hypoxia) and mechanoreceptors indicates an inability to respond adequately to the outgoing drive to breath, dyspnea occurs. Several lines of indirect evidence support the contention that hypoxia generates dyspnea *via* CD to higher centers if ventilation and PCO_2_ are constrained to normal levels (Moosavi et al., 2003, 2004).

If the pathophysiological mechanisms of the development of dyspnea are still poorly understood, we should not be surprised by our limited knowledge of the mechanisms of dyspnea in COVID-19 disease. From the physiopathology perspective, which does not exclude the direct neurotoxic effect of the virus and a systemic response in the infectious context, but rather encompasses it, the lack of dyspnea in the COVID-19 disease can be explained by matching the sensory cortex of the brain of the two signals coming from the motor command and periphery *via* CD.

## Author Contributions

The author confirms being the sole contributor of this work and has approved it for publication.

## Conflict of Interest

The author declares that the research was conducted in the absence of any commercial or financial relationships that could be construed as a potential conflict of interest.

## Publisher's Note

All claims expressed in this article are solely those of the authors and do not necessarily represent those of their affiliated organizations, or those of the publisher, the editors and the reviewers. Any product that may be evaluated in this article, or claim that may be made by its manufacturer, is not guaranteed or endorsed by the publisher.
